# Choroidal vessel density in unilateral hyperopic amblyopia using en-face optical coherence tomography

**DOI:** 10.1186/s12886-020-01735-z

**Published:** 2020-12-02

**Authors:** Syunsuke Araki, Atsushi Miki, Katsutoshi Goto, Tsutomu Yamashita, Tsuyoshi Yoneda, Atsushi Fujiwara, Kazuko Haruishi, Yoshiaki Ieki, Junichi Kiryu, Goro Maehara, Kiyoshi Yaoeda

**Affiliations:** 1 Department of Ophthalmology, Kawasaki Medical School, 577 Matsushima, Kurashiki, Okayama, 701-0192 Japan; 2 Doctoral Program in Sensory Science, Graduate School of Health Science and Technology, Kawasaki University of Medical Welfare, 288 Matsushima, Kurashiki, Okayama, 701-0193 Japan; 3 Department of Sensory Science, Faculty of Health Science and Technology, Kawasaki University of Medical Welfare, 288 Matsushima, Kurashiki, Okayama, 701-0193 Japan; 4 Division of Ophthalmology and Visual Sciences, Niigata University Graduate School of Medical and Dental Sciences, 1-757 Asahimachi-dori, Chuo-ku, Niigata, Niigata 951-8510 Japan; 5 Field of Orthoptics and Visual Sciences, Major in Medical and Rehabilitation Sciences, Graduate School of Health and Welfare, Niigata University of Health and Welfare, 1398 Shimami-cho, Kita-ku, Niigata, Niigata 950-3198 Japan; 6 Department of Human Sciences, Kanagawa University, 3-27-1 Rokkakubashi, Yokohama, Kanagawa 221-8686 Japan; 7 Yaoeda Eye Clinic, 2-1649-1 Naga-Chou, Nagaoka, Niigata 940-0053 Japan

**Keywords:** Amblyopia, Choroid, Vascular density, Optical coherence tomography

## Abstract

**Background:**

Structural changes of the choroid, such as choroidal thickening, have been indicated in amblyopic eyes with hyperopic anisometropia as compared to fellow or healthy eyes. The purpose of the present study was to investigate choroidal vascular density (CVD) in children with unilateral hyperopic amblyopia.

**Methods:**

This study included 88 eyes of 44 patients with unilateral amblyopia due to hyperopic anisometropia with or without strabismus and 29 eyes of 29 age-matched normal controls. The CVD of Haller’s layer was quantified from en-face images constructed by 3-dimensional swept-source optical coherence tomography images flattened relative to Bruch’s membrane. The analysis area was a 3 × 3-mm square of macula after magnification correction. Relationships between CVD and other parameters [best-corrected visual acuity (BCVA), refractive error and subfoveal choroidal thickness (SFCT)] were investigated, and CVDs were compared between amblyopic, fellow, and normal control eyes.

**Results:**

Mean CVD was 59.11 ± 0.66% in amblyopic eyes, 59.23 ± 0.81% in fellow eyes, and 59.29 ± 0.74% in normal control eyes. CVD showed a significant positive relationship with SFCT (*p* = 0.004), but no relationships with other parameters. No significant differences in CVD were evident among amblyopic, fellow, and normal control eyes after adjusting for SFCT (*p* = 0.502).

**Conclusions:**

CVD was unrelated to BCVA, and CVD did not differ significantly among amblyopic, fellow and normal control eyes. These results suggest that the local CVD of Haller’s layer is unaffected in unilateral hyperopic amblyopic eyes.

## Background

Amblyopia is defined as a disorder with dysfunctional processing of visual information, such as reduced recognition visual acuity (VA), resulting from either disuse due to the absence of a clear image on the retina or misuse due to abnormal binocular interactions [1]. Traditionally, morphological abnormalities have not been considered present in the ocular structure of amblyopic eyes. In contrast, recent studies using optical coherence tomography (OCT) have shown structural changes to the choroid such as thickening in hyperanisometropic amblyopic eyes compared with that of fellow or healthy eyes, independent of axial length (AL) [2–5]. In chorioretinal diseases such as central serous chorioretinopathy [6] and Vogt-Koyanagi-Harada disease [7], the pathogenesis and clinical significance of choroidal structural changes is relatively well understood, and OCT findings are utilized for diagnosis and follow-up. In contrast, no consensus has been reached regarding the pathogenesis or clinical significance of choroidal structural changes in patients with amblyopia.

Recently, a new method other than thickness has been developed to evaluate choroidal structures, focusing on choroidal vasculature using binarization analyses [8, 9]. Some studies have suggested changes in choroidal vasculature in amblyopic eyes, but insufficient knowledge has been accumulated [10–13]. The purpose of the present study was thus to investigate the clinical significance of evaluating choroidal vascular density (CVD) in patients with amblyopia.

## Methods

All investigative procedures adhered to the tenets of the Declaration of Helsinki. This study was approved by the Institutional Review Board Committee at Kawasaki Medical School (registration number: 3473). This study was designed as a retrospective observational case series and was conducted from November 2013 to December 2018 in the Department of Ophthalmology at Kawasaki Medical School Hospital. This study included minors under 16 years old among the patient sample. Informed consent for all examinations was obtained from one parent of each patient.

### Subjects

Patients enrolled to this study showed unilateral amblyopia with decimal best-corrected visual acuities (BCVAs) < 0.8 in the amblyopic eye and > 1.0 in the fellow eye at the time of the first visit, due to hyperopic anisometropia with or without strabismus; and age ≦ 18 years. Hyperopic anisometropia was defined as hypermetropia in both eyes and an interocular difference in refraction (spherical equivalent) of > 1.5 diopters (D). Treatment status was not considered at the time of performing swept-source OCT (SS-OCT) in this study. Exclusion criteria were as follows: presence of decimal BCVA in amblyopic eyes > 1.0 within 2 months after full refractive correction or at the time of SS-OCT; history of ocular disease; history of intraocular surgery; or presence of systemic diseases that may have exerted an influence on the eye.

A total of 29 children were recruited as a normal control group. These children in the control group had a decimal BCVA > 1.0 in both eyes, and no ocular or systemic diseases other than mild refractive errors. Only the right eye of the control group was used for data analysis.

Ophthalmologic examinations performed in all subjects included BCVA, cycloplegic refraction (measured using a RKT-7700 auto-refractor; NIDEK, Gamagori, Japan), AL (measured using an IOL Master OCT biometer; Carl Zeiss Meditec AG, Jena, Germany), cover test, extraocular movements, slit-lamp, fundoscopy, and SS-OCT (DRI OCT-1 Atlantis; Topcon Corporation, Tokyo, Japan). All SS-OCT examinations were performed by the same author (S.A.) between 9:00 AM and 12:00 PM under nonmydriatic conditions.

### Quantification of choroidal vascular density and subfoveal choroidal thickness

**Fig. 1:**
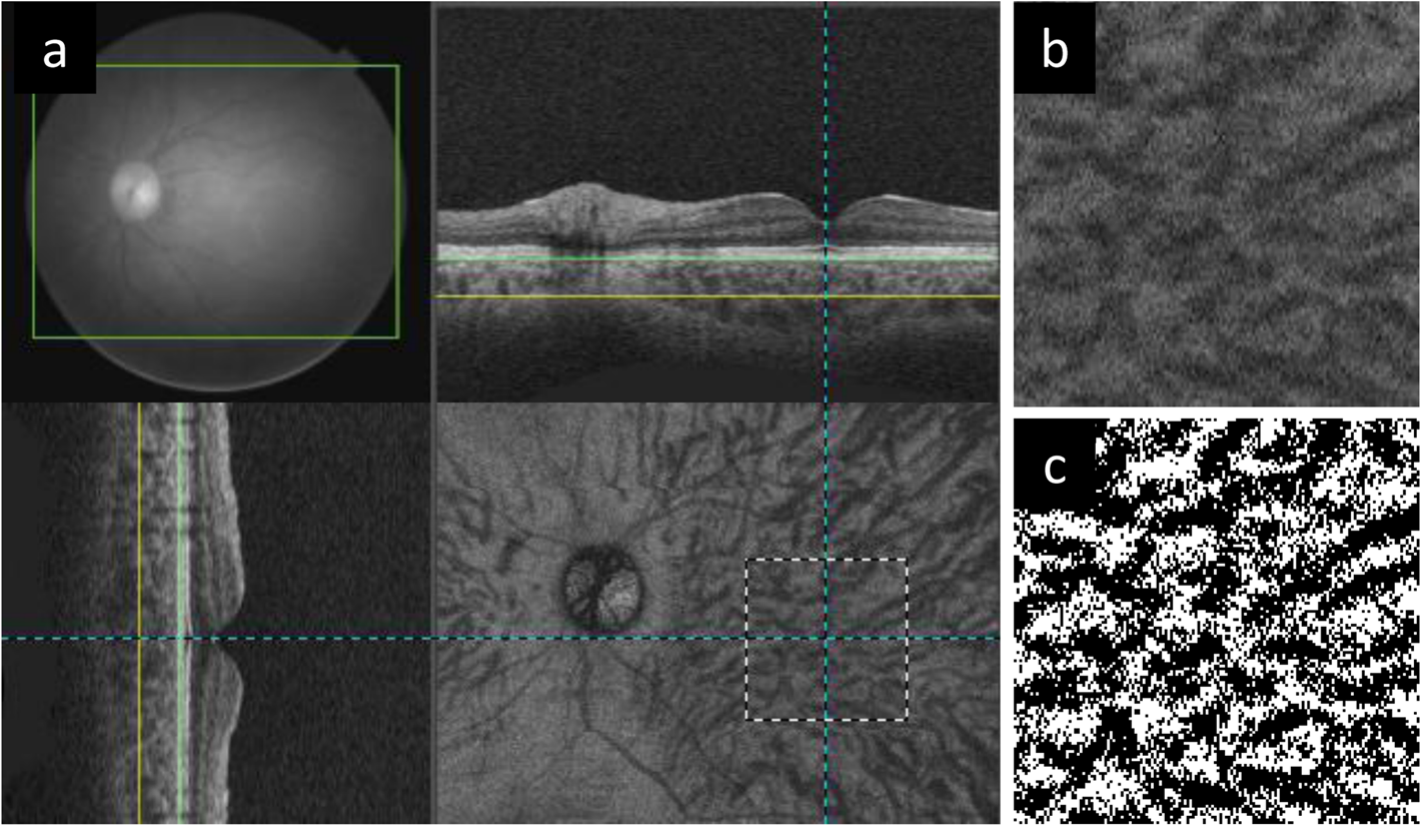
Analysis of choroidal vascular density using binarized en-face images. **a** Three-dimensional swept-source optical coherence tomography images were reconstructed as en-face images flattened with Bruch’s membrane. The yellow line shows where the subfoveal choroidal thickness is 50% level of total thickness. The white dotted-line shows the 3 × 3-mm square centered on the fovea after correction for magnification error using individual axial length. **b** Extracted 3 × 3-mm square en-face image. **c** En-face image after binarization. The black area is considered to be the choroidal vascular area, and the percentage within the region of interest is calculated as the choroidal vascular density

CVD was quantified by binarizing the en-face image with reference to the report by Fujiwara et al. [9]. The 3-dimensional (3D) SS-OCT images, which covered a 12 × 9-mm^2^ area with a scan density of 512 × 256, were reconstructed as en-face images flattened with Bruch’s membrane using dedicated software tools (EnView version 1.0.1; Topcon Corporation). En-face images were extracted at the level where the distance from the inner surface of the choroid was 50% of the total subfoveal choroidal thickness (SFCT) to examine the CVD of Haller’s layer (Fig. 1a).

The analyzed region of CVD was a 3 × 3-mm square centered on the fovea (Fig. 1b). The magnification error was corrected using the Littman and modified Bennett formulae, taking into account image magnification due to AL variation [14, 15]. The extracted images were binarized by Niblack’s method using ImageJ software (version 1.51; National Institutes of Health, Bethesda, MD), and the percentage of black area (considered to represent the choroidal vascular area) in the total region of interest was calculated as CVD (Fig. 1c).

SFCT was defined as the vertical distance from the outer border of the hyperreflective line of retinal pigment epithelium/Bruch’s complex to the choroidal-scleral juncture at the subfovea. The SFCT was measured manually by S.A. from 3D SS-OCT images using the built-in caliper tool. Subjects with images that were difficult to segment due to signal attenuation were excluded from the present study.

### Statistical analyses

Data are presented as means ± standard deviations. For statistical analysis, the decimal BCVA was converted to the logarithm of the minimal angle of resolution (logMAR).

First, multiple regression analysis of all eyes using the forced entry method was performed with CVD as a dependent variable, and logMAR, refractive error (RE), and SFCT as independent variables to select those parameters to be corrected for comparisons of CVD. Because AL and RE are highly collinear and mutually confounding variables [16], only RE was used for the multiple regression analyses.

Second, a generalized linear mixed model (GLMM) was used for CVD comparisons with measured eye (amblyopic, fellow, or normal control eyes) as a fixed factor, individual subjects (amblyopic patients or healthy control children) and factors that were significant in multiple regression analysis as random factors. In addition, logMAR, RE, AL, and SFCT were compared among amblyopic, fellow and normal control eyes. If a significant difference was found by GLMM, pairwise comparison was performed with Bonferroni correction.

The two-sample t-tests and chi-squared test were used to compare age and sex ratio between patients with unilateral amblyopia and normal control groups, respectively. Pearson’s correlation analysis was used to determine the coefficient of correlation between CVD and logMAR in amblyopic eyes.

A value of *p* <  0.05 was considered statistically significant. All statistical analyses were conducted using SPSS Statistics version 20.0 (IBM Corporation, Somers, New York, USA).

## Results

### Demographic data

This study enrolled 44 patients with unilateral hyperopic amblyopia and 29 normal control individuals. All subjects were Japanese. Table 1 shows demographic and clinical data for all subjects. In the amblyopic group, mean age at the time of SS-OCT was 6.8 ± 3.0 y (range, 3–18 y), and 29 patients (66%) were female. In the normal control group, mean age at the time of SS-OCT was 7.6 ± 3.1 y (range, 3–16 y), and 18 (62%) individuals were female. No significant differences were evident between amblyopic and normal control groups with regard to age (*p* = 0.23) or sex ratio (*p* = 0.74).

**Table 1:** Demographic and clinical data at SS-OCT measurements

	AE (*n* = 44)	FE (*n* = 44)	Cont. (*n* = 29)	*p*-value (AE vs FE)	*p*-value (AE vs Cont.)	*p*-value (FE vs Cont.)
Age	6.8 ± 3.0		7.6 ± 3.1	–	–	–
Sex (Male: Female)	15: 29		11: 18	–	–	–
Visual acuity (logMAR)	0.38 ± 0.22	− 0.13 ± 0.07	−0.12 ± 0.07	< 0.001^a^	< 0.001^a^	1.000^a^
Refractive error (diopters)	5.32 ± 1.70	1.68 ± 1.31	0.46 ± 1.44	< 0.001^a^	< 0.001^a^	0.003^a^
Axial length (mm)	21.00 ± 0.98	22.29 ± 1.06	22.77 ± 1.11	< 0.001^a^	< 0.001^a^	0.170^a^
Subfoveal choroidal thickness (μm)	387.6 ± 78.4	298.3 ± 42.5	323.8 ± 69.1	< 0.001^b^	< 0.001^b^	0.302^b^

Values are shown as mean ± standard deviation

*AE* amblyopic eyes, *FE* fellow eyes, *Cont.* normal control eyes

^a^Bonferroni correction after GLMM

^b^Bonferroni correction after GLMM corrected with axial length

Mean BCVA (logMAR) in amblyopic eyes was significantly worse than that of fellow or normal control eyes (*p* <  0.001, each), with no significant difference between fellow and normal control eyes (*p* = 1.000). Mean RE in amblyopic eyes was more hyperopic than that of fellow and normal control eyes, and was more hyperopic in the fellow eyes than in the normal control eyes (*p* <  0.001, *p* <  0.001, and *p* = 0.003, respectively). Mean AL in amblyopic eyes was significantly shorter than in fellow or normal control eyes (*p* <  0.001, each), with no significant difference between fellow and normal control eyes (*p* = 0.170). Mean SFCT was significantly thicker in amblyopic eyes than in fellow or normal control eyes (*p* <  0.001, each ), with no significant difference between fellow and normal control eyes (*p* = 0.302).

### CVD

**Fig. 2:**
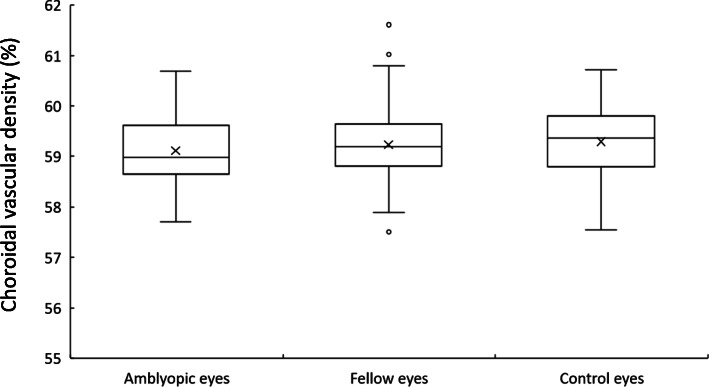
Box plot of the choroidal vascular density in amblyopic, fellow and normal control eyes. Cross symbols show the mean values. Circle symbols show the outlier values which are more than 1.5 box lengths apart from either end of the box. There was no significant difference in the choroidal vascular density among the amblyopic, fellow and normal control eyes with or without correction for subfoveal choroidal thickness (amblyopic eyes vs. fellow eyes, *p* = 1.000, *p* = 1.000; amblyopic eyes vs. control eyes, *p* = 0.990, *p* = 0.963; fellow eyes vs. control eyes, *p* = 1.000, *p* = 1.000; Bonferroni correction)

**Fig. 3:**
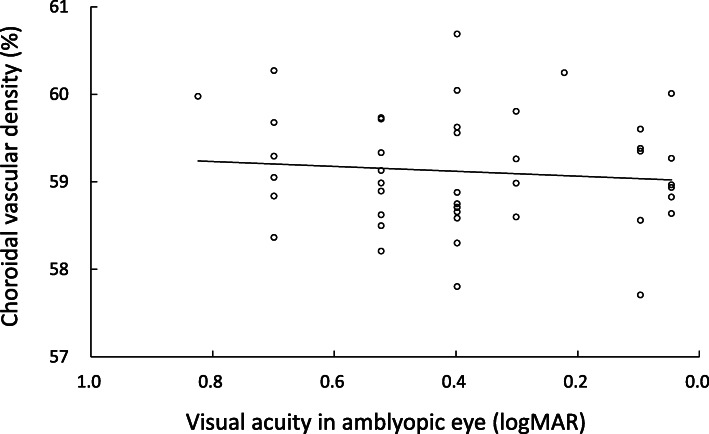
Relationship between choroidal vascular density and best-corrected visual acuity in amblyopic eyes. No significant correlation is evident between choroidal vascular density and best-corrected visual acuity (*r* = 0.094, *p* = 0.545)

Mean CVD was 59.11 ± 0.66% in amblyopic eyes, 59.23 ± 0.81% in fellow eyes and 59.29 ± 0.74% in normal control eyes (Fig. 2). Table 2 shows the results of multiple regression analysis of all eyes for CVD. SFCT was significantly associated with CVD (standardized β = 0.307, *p* = 0.004). logMAR and RE were not associated factors with CVD. No significant difference in mean CVD was seen among amblyopic, fellow or normal control eyes with or without correction for SFCT (*p* = 0.502, *p* = 0.505, respectively; GLMM). In amblyopic eyes, no significant correlation existed between CVD and logMAR (*r* = 0.094, *p* = 0.545) (Fig. 3).

**Table 2:** Multiple regression analysis of all eyes for choroidal vascular density (*n* = 117)

Independent variable	Standardized β	Partial correlation coefficient	*p*-value
LogMAR	−0.113	−0.071	0.449
Refractive error	−0.169	−0.109	0.247
Subfoveal choroidal thickness	0.307	0.265	0.004

## Discussion

The current findings showed that the CDV of Haller’s layer, as analyzed by en-face OCT, did not differ significantly among amblyopic, fellow and normal control eyes. Also, no significant correlation was apparent between CVD and BCVA in amblyopic eyes.

The choroidal structure has been implicated in the pathogenesis of various ocular diseases. Agrawal et al. recently reported that choroidal vascularity index (CVI: the ratio of luminal area to total choroid area, equivalent to CVD) is a robust marker for studying pathophysiologies of the choroid, because CVI is less variable than SFCT and is not associated with most physiological variables [17]. In fact, studies in patients with Stargardt disease [18] and retinopathy of prematurity [19] have found that CVI is a more sensitive biomarker than SFCT and that a decrease in CVI is associated with a decrease in BCVA. On the other hand, the present study observed no specific changes in the CVD of amblyopic eyes, and no correlation with BCVA, suggesting that CVD may not be an adequate biomarker for evaluating visual acuity loss in amblyopic patients. However, visual dysfunction in amblyopia can be evaluated using many parameters other than visual acuity. For example, amblyopic eyes show poorer accommodative performance than fellow or normal eyes [20]. Accommodation has been reported directly related to choroidal structure [21]. Therefore, novel findings may be obtained by using indices other than BCVA (e.g., accommodation) in the investigation of the relationship between amblyopia and CVD in the future.

Several reports have examined choroidal vascular structures in patients with amblyopia [10–13]. In reports using analyses by binarization of a single-section OCT image, Nishi et al. found that the luminal area was significantly larger and the stromal area was significantly smaller in amblyopic eyes than in control hyperopic eyes [10]. Another study showed a reduction in the luminal area and widened stromal area after treatment only in amblyopic eyes [13]. Beak et al. reported that the ratio of choroidal luminal area to total choroidal area (i.e., CVD) of amblyopic eyes showed different characteristics from normal control eyes, such as amblyopic eyes showing higher CVD that did not increase in direct proportion to the increased choroidal thickness [12]. In contrast to those previous reports [10, 12, 13], the present study did not find any difference in CVD between amblyopic patients and normal healthy subjects. Our study extracted en-face images at a fixed distance from Burch’s membrane and analyzed the CVD. On the other hand, CVD analysis using B-scan images, as used in previous studies [10, 12, 13], is able to analyze only one cross-section image, while allowing analysis of the structure of the inner and outer layers of choroid simultaneously. Differences in results between these studies may thus be due to differences in the methods used for CVD analysis.

On the basis of analyzing binarized en-face OCT images, Terada et al. reported that the outer choroidal vascular areas (i.e., CVD of Haller’s layer) were larger in both amblyopic (61.49 ± 4.95%) and fellow eyes (61.48 ± 3.73%) than in healthy eyes (55.69 ± 1.83%) [11]. They also indicated that the cutoff for distinguishing between amblyopic patients and controls was 59%. In our current study, the CVD in amblyopic and fellow eyes exceeded 59% (59.11 ± 0.66% and 59.23 ± 0.81%, respectively), consistent with a report by Terada et al. However, the CVD in normal control eyes (59.29 ± 0.74%) also exceeded 59%, resulting in comparable CVDs among different eye conditions. In normal eyes, CVD has been reported to correlate positively with SFCT [9]. However, SFCT was slightly thicker in Terada’s control group (351.9 ± 60.7 μm) than in our control group (323.8 ± 69.1 μm). The higher CVD in the normal control eye is thus not attributable to differences in SFCT. Substantial individual differences might be present in CVD. We should therefore be cautious of using CVD analysis of en-face OCT images to evaluate amblyopia.

The present study showed that SFCT was significantly thicker in the amblyopic eye than in fellow and normal control eyes, while CVD was comparable among all eyes. The results indicate that choroidal blood flow with respect to total choroidal volume may be increased in amblyopic eyes than in fellow and normal control eyes. A possible future direction of this work would be to investigate the ratio of choroidal vessels to total volume of the choroid in amblyopic eyes.

Several limitations should be considered when interpreting the findings of the present study. First, the sample size was small. A further study of a larger number of patients with detailed classifications of the degree and type of amblyopia will need to be undertaken to validate our findings. Second, CVD could only be assessed in a 3 × 3-mm localized area centered on the fovea. This was due to the large difference in thickness between the center and periphery of the choroid, making it difficult to capture the same depth of vascular structure in the center and periphery if the analyzed area of the en-face image flattened with Bruch’s membrane is expanded. In the future, new tools, such as high-penetration doppler optical coherence angiography [22] and hybrid three-dimensional models of the choroidal vasculature [23], are expected to provide a more detailed assessment of choroidal vascular structures.

## Conclusions

In conclusion, the CVD of Haller’s layer did not differ significantly between amblyopic eyes and fellow or normal control eyes. Evaluation of CVD using en-face OCT may have little clinical significance in elucidating the mechanisms underlying amblyopia.

## Data Availability

The datasets used and/or analysed during the current study are available from the corresponding author on reasonable request.

## Abbreviations

AL: Axial length
BCVA: Best-corrected visual acuity
CVD: Choroidal vascular density
CVI: Choroidal vascularity index
GLMM: Generalized linear mixed model
logMAR: Logarithm of the minimal angle of resolution
SFCT: Subfoveal choroidal thickness
SS-OCT: Swept-source optical coherence tomography
RE: Refractive error
